# Cell aggregation activates small GTPase Rac1 and induces CD44 cleavage by maintaining lipid raft integrity

**DOI:** 10.1016/j.jbc.2023.105377

**Published:** 2023-10-20

**Authors:** Dong Li, Younhee Park, Hami Hemati, Xia Liu

**Affiliations:** 1 Department of Toxicology and Cancer Biology, College of Medicine, University of Kentucky, Lexington, Kentucky, USA; 2 Markey Cancer Center, University of Kentucky, Lexington, Kentucky, USA

**Keywords:** cell aggregation, lipid raft, CD44 ICD, Rac1, breast cancer, metastasis, circulating tumor cells, anoikis

## Abstract

Lipid rafts are highly ordered membrane domains that are enriched in cholesterol and glycosphingolipids and serve as major platforms for signal transduction. Cell detachment from the extracellular matrix (ECM) triggers lipid raft disruption and anoikis, which is a barrier for cancer cells to metastasize. Compared to single circulating tumor cells (CTCs), our recent studies have demonstrated that CD44-mediatd cell aggregation enhances the stemness, survival and metastatic ability of aggregated cells. Here, we investigated whether and how lipid rafts are involved in CD44-mediated cell aggregation. We found that cell detachment, which mimics the condition when tumor cells detach from the ECM to metastasize, induced lipid raft disruption in single cells, but lipid raft integrity was maintained in aggregated cells. We further found that lipid raft integrity in aggregated cells was required for Rac1 activation to prevent anoikis. In addition, CD44 and γ-secretase coexisted at lipid rafts in aggregated cells, which promoted CD44 cleavage and generated CD44 intracellular domain (CD44 ICD) to enhance stemness of aggregated cells. Consequently, lipid raft disruption inhibited Rac1 activation, CD44 ICD generation, and metastasis. Our findings reveal two new pathways regulated by CD44-mediated cell aggregation *via* maintaining lipid raft integrity. These findings also suggest that targeting cell aggregation-mediated pathways could be a novel therapeutic strategy to prevent CTC cluster-initiated metastasis.

Metastasis is responsible for the majority of cancer-related patient deaths. To metastasize, cancer cells have to detach from the primary tumor and travel to different sites. Usually, cells undergo apoptosis after losing contact with their extracellular matrix (ECM), which has been termed “anoikis” and is a barrier for tumor cells to metastasize (1). However, some cancer cells can develop mechanisms to resist anoikis and survive after detachment from their primary site, giving rise to metastasis (2, 3, 4, 5, 6). Thus, a better understanding of the mechanism underlying anoikis resistance of cancer cells after detachment will provide the basis for developing therapeutics to eliminate metastasizing cancer cells.

Lipid rafts are highly ordered membrane domains that are enriched in cholesterol and glycosphingolipids and serve as major platforms for signal transduction (7, 8, 9). It has been reported that cell detachment from the ECM triggers the internalization of lipid rafts (or lipid raft disruption) and anoikis (1, 10). Recently, we found that CD44-mediated cell aggregation prevents cells from anoikis after cell detachment (11, 12). We also found that CD44-mediated cell aggregation activates Pak2 (p21-activated kinase 2) (11, 12), a downstream target of Rac1 (13). Previous studies have shown that lipid raft integrity is required for maintaining Rac1 membrane targeting and effector activation in ECM-detached cells (14). Furthermore, Rac1 activation can prevent epithelial cells from anoikis (15, 16). Taken together, it raises a possibility that CD44-mediated cell aggregation may maintain lipid raft integrity to activate Rac1-Pak2 pathway to prevent anoikis.

CD44 is a well-known breast cancer stem cell marker (17, 18). Proteolytic cleavage of CD44 is a regulatory mechanism for the CD44-mediated signaling pathways (19, 20). CD44 is cleaved by matrix metallopeptidases to generate the membrane-bound C-terminal fragment of CD44 (CD44 EXT), followed by γ-secretase to generate CD44 intracellular domain (CD44 ICD) (19, 20, 21, 22, 23, 24). CD44 ICD then is translocated to the nucleus as a signal transduction molecule, and activates gene transcription, including stemness factors such as Oct4, to enhance cell stemness and promote tumorigenesis (24, 25, 26). Both CD44 and γ-secretase are present in lipid rafts (27, 28, 29), and we have found that aggregated CD44^+^ triple-negative breast cancer (TNBC) cells have enhanced stemness by upregulating Oct4 (11), suggesting lipid rafts may be involved in enhanced stemness of CD44-mediated cell aggregation.

In this study, we discovered that CD44-mediated cell aggregation maintains lipid raft integrity after cell detachment, which then activates Rac1 to prevent anoikis of detached cells, and generates CD44 ICD to enhance stemness of aggregated cells *via* promoting CD44 cleavage. These findings provide a potential therapeutic strategy to prevent CTC cluster-initiated metastasis by blocking cell aggregation-mediated downstream pathways.

## Results

### Cell aggregation maintains lipid raft integrity after cell detachment

**Figure 1:**
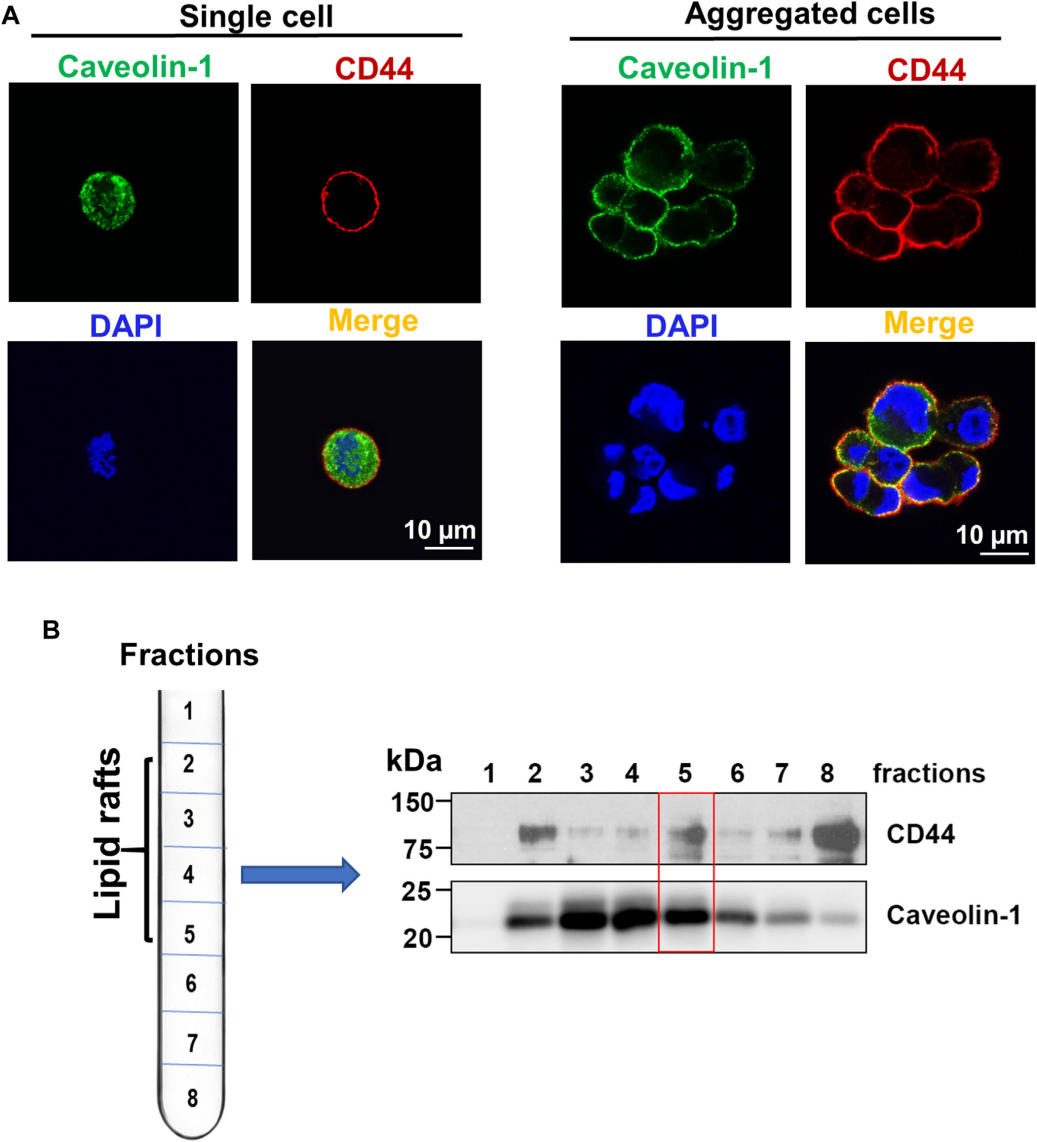
**CD44-mediated cell aggregation maintains lipid raftintegrity after cell detachment.***A*, immunofluorescence staining shows that CD44-mediated cell aggregation maintains lipid rafts integrity in aggregated cells (*right*), but not the single cells (*left*) after cell detachment. The MDA-MB-231 cells were cultured in poly-HEMA-coated dishes to aggregate for 24 h, and then cells were dried onto cover slides for IF staining. *Red*, CD44; *Green*, caveolin-1 (lipid raft marker); *Blue*, DAPI. *B*, CD44 colocalizes with caveolin-1 at lipid rafts. The MDA-MB-231 cells were cultured in poly-HEMA-coated dishes to aggregate for 24 h, and then lipid rafts were isolated using Caveolae/Rafts Isolation Kit (the lipid rafts are enriched in 2–5 fractions showing high caveolin-1 expression). The co-localization of CD44 with caveolin-1 in fraction 5 was indicated in the *red box*. The data represent one of three independent experiments.

Cell detachment changes the structure of the cell membrane and affects the activities of many downstream signaling pathways. Lipid rafts are highly ordered membrane domains that serve as major platforms for signal transduction (7, 8, 9). We cultured MDA-MB-231 cells in the poly-HEMA-coated dishes to prevent cell adhesion and force them to grow in suspension, which mimics the condition after tumor cells are detached from the ECM to metastasize. We found that caveolin-1 (the marker for lipid rafts) is delocalized from the cell membrane into the cytoplasm, suggesting that lipid rafts are disrupted in the single cells (Fig. 1*A*). However, the lipid raft integrity (caveolin-1 mainly localizes on the cell membrane) was maintained in aggregated CD44^+^ cells (Fig. 1*A*). In addition, we found that CD44 colocalizes with caveolin-1 on cell membranes in aggregated cells (Fig. 1*A*). Since caveolin-1 is a marker for lipid rafts, we further determined whether CD44 colocalizes with caveolin-1 in lipid rafts. We isolated lipid rafts from aggregated MDA-MB-231 cells after suspension culture for 24 h and found that CD44 was highly expressed and coexisted with caveolin-1 in fraction 5 (according to the instruction of the isolation kit, the lipid rafts are enriched in 2 to 5 fractions, Fig. 1*B*), suggesting that CD44 localizes at lipid rafts in the aggregated cells. Collectively, these data suggest that cell detachment induces lipid raft disruption in single cells, but cell aggregation maintains lipid raft integrity after cell detachment.

### Disruption of lipid rafts induces aggregated cells to undergo anoikis

**Figure 2:**
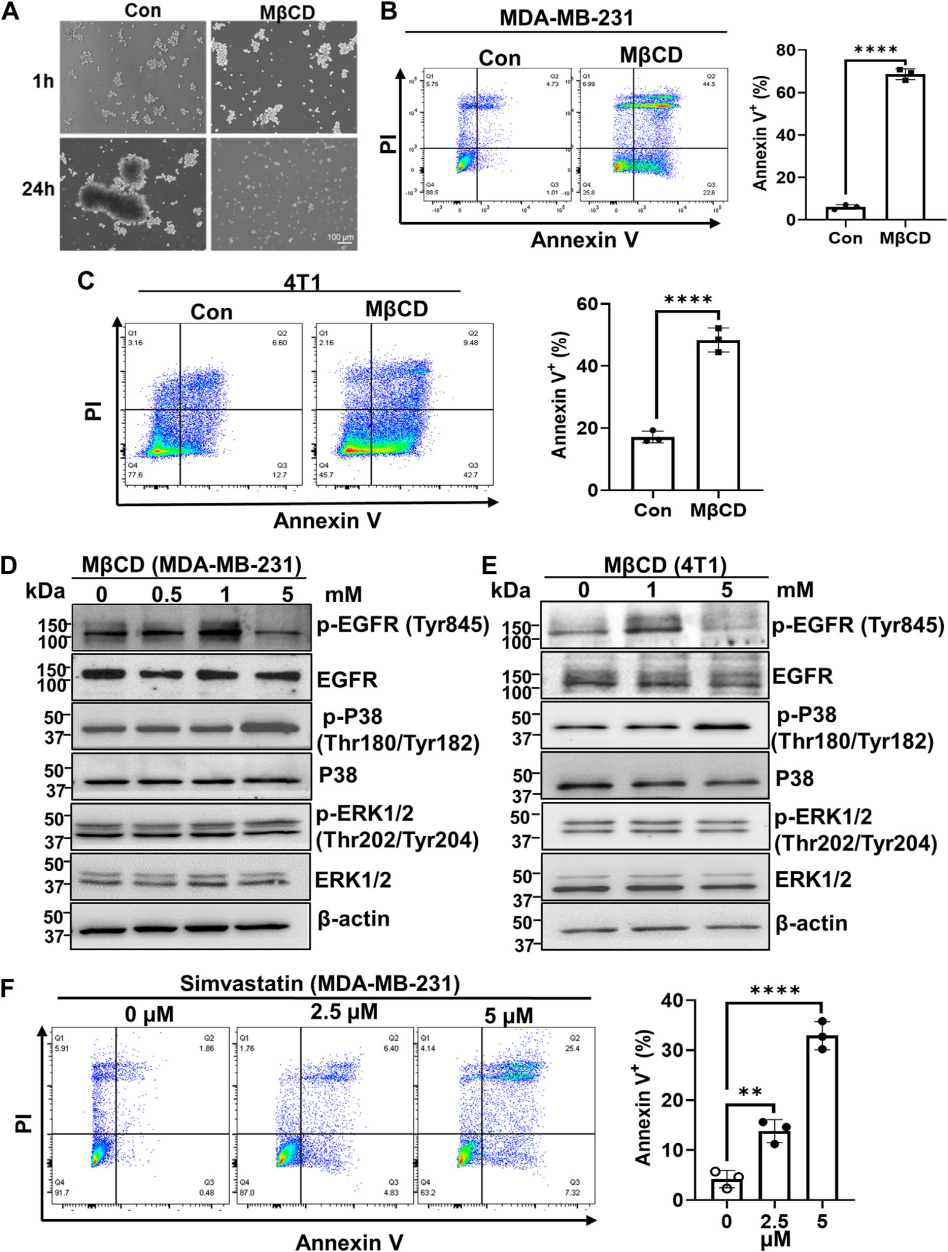
**Disruption of lipid rafts promotes aggregated cells to undergo anoikis.***A*, representative images of MDA-MB-231 cells cultured in poly-HEMA-coated dishes in the absence or presence of MβCD (5 mM, pretreatment for 1 h) for the indicated time points. *B* and *C*, disruption of lipid rafts by MβCD promotes anoikis. The MDA-MB-231 cells (*B*) and 4T1 cells (*C*) were cultured in poly-HEMA-coated dishes in the presence or absence of MβCD (5 mM) for 24 h, and then the cells were collected for anoikis analysis by Annexin V/PI staining (*left*). The percentage of Annexin V^+^ cells was quantitated (*right*). Graph data were presented as mean ± SD (n = 3). *t-*test, ∗∗∗∗*p* < 0.0001. *D* and *E*, Western blotting analysis of p-EGFR, p-p38 and p-ERK expression in MDA-MB-231 cells (*D*) and 4T1 cells (*E*) cultured in poly-HEMA-coated dishes in the presence or absence of MβCD for 24 h. β-actin serves as a loading control. The data represent one of two independent experiments. *F*, simvastatin induces anoikis. The MDA-MB-231 cells were cultured in poly-HEMA-coated dishes in the presence or absence of simvastatin for 48 h, and the cells were collected for anoikis analysis by Annexin V/PI staining. The percentage of Annexin V^+^ cells was quantitated (*right*). Graph data were presented as mean ± SD (n = 3). One-way ANOVA, ∗∗*p* < 0.01, ∗∗∗∗*p* < 0.0001.

Normally, cells undergo anoikis after they lose contact with ECM. However, we previously found that CD44-mediated cell aggregation can prevent anoikis after cell detachment (11). Since we found that cell aggregation maintains lipid raft integrity (Fig. 1), and studies have demonstrated that lipid raft integrity is required for the survival of TNBC cells (30), we examined whether disruption of lipid rafts could induce anoikis in aggregated cells. We pretreated MDA-MB-231 cells for 1 h with or without MβCD (5 mM, at the dosage which is sufficient to deplete the cholesterol in both MDA-MB 231 and 4T1 cells (31)), a commonly used reagent to disrupt lipids rafts by depletion of cholesterol (32) and then cultured them in the poly-HEMA-coated dishes to allow them to aggregate. Although cell aggregation was not changed by MβCD (Fig. 2*A*), the anoikis, which was determined by Annexin V/PI staining, was significantly increased in MβCD-treated cells at 24 h (Fig. 2*B*). The similar results were obtained in the 4T1 TNBC cell line (Fig. 2*C*). These data suggest that lipid raft disruption promotes aggregated cells to undergo anoikis.

Previously, we have shown that cell aggregation activates EGFR (33), which plays an important role in preventing cells from anoikis. Consistently, MβCD (5 mM) treatment reduced EGFR activation (indicated by the phosphorylation of EGFR at Y845) in both detached MDA-MB-231 and 4T1 cells (Fig. 2, *D* and *E*). It was reported that ERK activation by EGFR is required for preventing anoikis in Her2-positive breast cancer cells (34). However, the ERK activity (determined by an active form of phosphorylated ERK) was not changed by lipid raft disruption in both MDA-MB-231 and 4T1 TNBC cells (Fig. 2, *D* and *E*). Instead, p38 mitogen-activated protein kinase activity was increased in MβCD-treated cells (Fig. 2, *D* and *E*). It has been reported that p38 activation induces anoikis (35, 36). To investigate whether p38 activation plays a role in lipid rafts disruption-induced anoikis, cells were preincubated with p38 inhibitor for 1 h and then cultured in poly-HEMA-coated dishes with or without MβCD (5 mM) for 24 h. The anoikis analysis indicated that inhibition of p38 can partially prevent anoikis triggered by lipid rafts disruption (Fig. S1). These data suggest that p38 activation is involved in lipid rafts disruption-induced anoikis. To further confirm that lipid rafts disruption promotes aggregated cells to undergo anoikis, we cultured MDA-MB-231 cells in the poly-HEMA-coated dishes in the absence or presence of simvastatin, which is another reagent that can disrupt lipid rafts by reducing the cholesterol content of lipid rafts (37). Similarly, simvastatin also promoted anoikis of aggregated cells (Fig. 2*F*). Taken together, these data indicate that disruption of lipid rafts induces aggregated cells to undergo anoikis.

### Lipid raft integrity is required for Rac1 activation in aggregated cells to prevent anoikis

**Figure 3:**
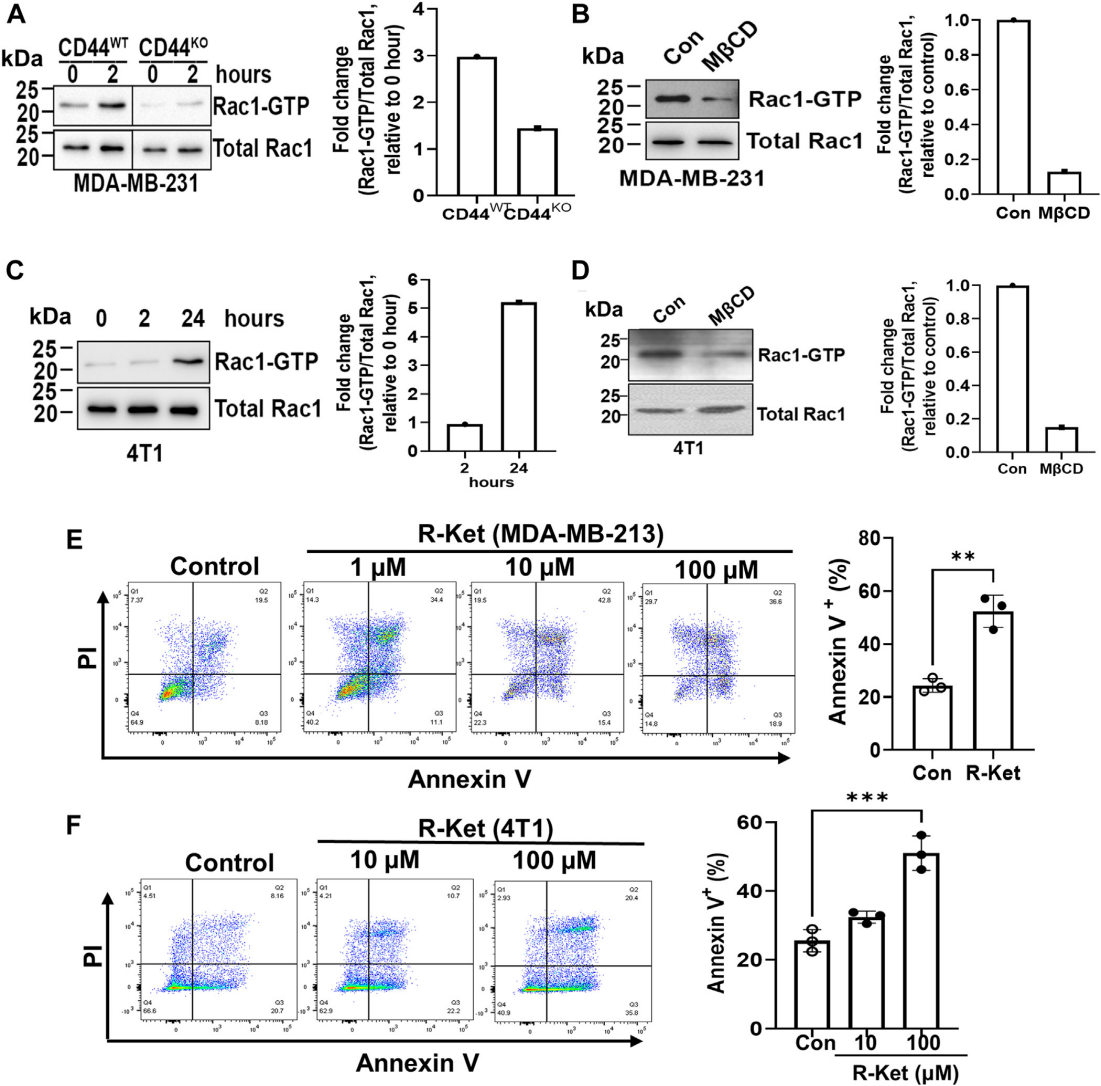
**Lipid raft integrity is required for Rac1 activation in aggregated cells to prevent anoikis.***A*, Rac1 activity in CD44 ^WT^ MDA-MB-231 cells is dramatically increased compared to CD44 ^KO^ MDA-MB-231 cells when cultured in poly-HEMA-coated dishes (*left*). The levels of active Rac1-GTP are normalized with the levels of total Rac1 protein in cells, and the fold changes compared with 0 h of each cell line are calculated (*right*). The data represent one of two independent experiments. *B*, lipid raft disruption (5 mM MβCD for 2 h) inhibits Rac1 activation in aggregated CD44^WT^ MDA-MB-231 cells. The levels of active Rac1-GTP are normalized with the levels of total Rac1 protein in cells, and the fold changes compared with control are calculated (*right*). The data represent one of two independent experiments. *C*, Rac1 activity in 4T1 cells is dramatically increased when cultured in poly-HEMA-coated dishes for 24 h to aggregate (*left*). The levels of active Rac1-GTP are normalized with the levels of total Rac1 protein in cells, and the fold changes compared with 0 h are shown (*right*). The data represent one of two independent experiments. *D*, lipid rafts disruption inhibits Rac1 activation in aggregated 4T1 cells. The levels of active Rac1-GTP are normalized with the levels of total Rac1 protein in cells, and the fold changes compared with control are calculated (*right*). The data represent one of two independent experiments. *E*, the MDA-MB-231 cells were cultured in poly-HEMA-coated dishes in the presence or absence of R-Ketorolac for 48 h, and the cells were collected for anoikis analysis by Annexin V/PI staining. Control and 1 μm R-Ketorolac (R-Ket)-treated groups were used for quantification, and graph data were presented as mean ± SD (n = 3). *t-*test, ∗∗*p* < 0.01. *F*, the 4T1 cells were cultured in poly-HEMA-coated dishes in the presence or absence of R-Ketorolac for 48 h, and the cells were collected for anoikis analysis by Annexin V/PI staining. Graph data were presented as mean ± SD (n = 3). One-way ANOVA, ∗∗∗*p* < 0.001.

Recently, we found that CD44-mediated cell aggregation activates Pak2 (11, 12), which is a downstream target of Rac1 (13). Therefore, we investigated whether CD44-mediated cell aggregation activates Rac1. Indeed, Rac1 activity was dramatically increased in CD44 wild-type (CD44^WT^), but not CD44 knockout (CD44^KO^) MDA-MB-231 cells after suspension culture in poly-HEMA-coated dishes for 2 h (Fig. 3*A*), suggesting that CD44-mediated cell aggregation activates Rac1. However, disruption of lipid raft integrity *via* MβCD inhibited Rac1 activation in aggregated CD44^WT^ MDA-MB-231 cells (Fig. 3*B*). Consistently, Rac1 activity was also increased in aggregated 4T1 cells after cultured in poly-HEMA-coated dishes for 24 h, but MβCD inhibited Rac1 activation (Fig. 3, *C* and *D*). These data suggest that lipid raft integrity is required for Rac1 activation in aggregated cells. Since Rac1 activation prevents anoikis (15, 16), and lipid raft integrity is required for maintaining Rac1 membrane targeting and effector activation in ECM-detached cells (14), we next examined the effect of Rac1 inhibition on anoikis. To this end, the cells were cultured in poly-HEMA-coated dishes, and treated in the absence or presence of racemic ketorolac (R-ketorolac), which has been identified as a robust Rac1 inhibitor at dosage above 10 μM (38, 39). We found that R-ketorolac significantly increased the anoikis (Annexin V^+^ cells) of aggregated cells (Fig. 3, *E* and *F*) without effect on cell aggregation after detachment (Fig. S2). It is worth noting that although higher dosage of R-ketorolac (10 μM and 100 μM) did not further increase Annexin V+ cells (Q2 + Q4) in MDA-MB-231 cells compared with lower dosage (1 μM), the single PI+ cells (dead cells) were increased in a dosage-dependent manner (14.3% at 1 μM, 19.5% at 10 μM and 29.7% at 100 μM, respectively), resulting in the significant reduction of live cells (Q4) in higher doses (40.2% at 1 μM, 22.3% at 10 μM and 14.8% at 100 μM, respectively). Collectively, these data indicate that lipid raft integrity is required for Rac1 activation in aggregated cells, contributing to anoikis resistance.

### CD44-mediated cell aggregation promotes CD44 cleavage to generate CD44 ICD

**Figure 4:**
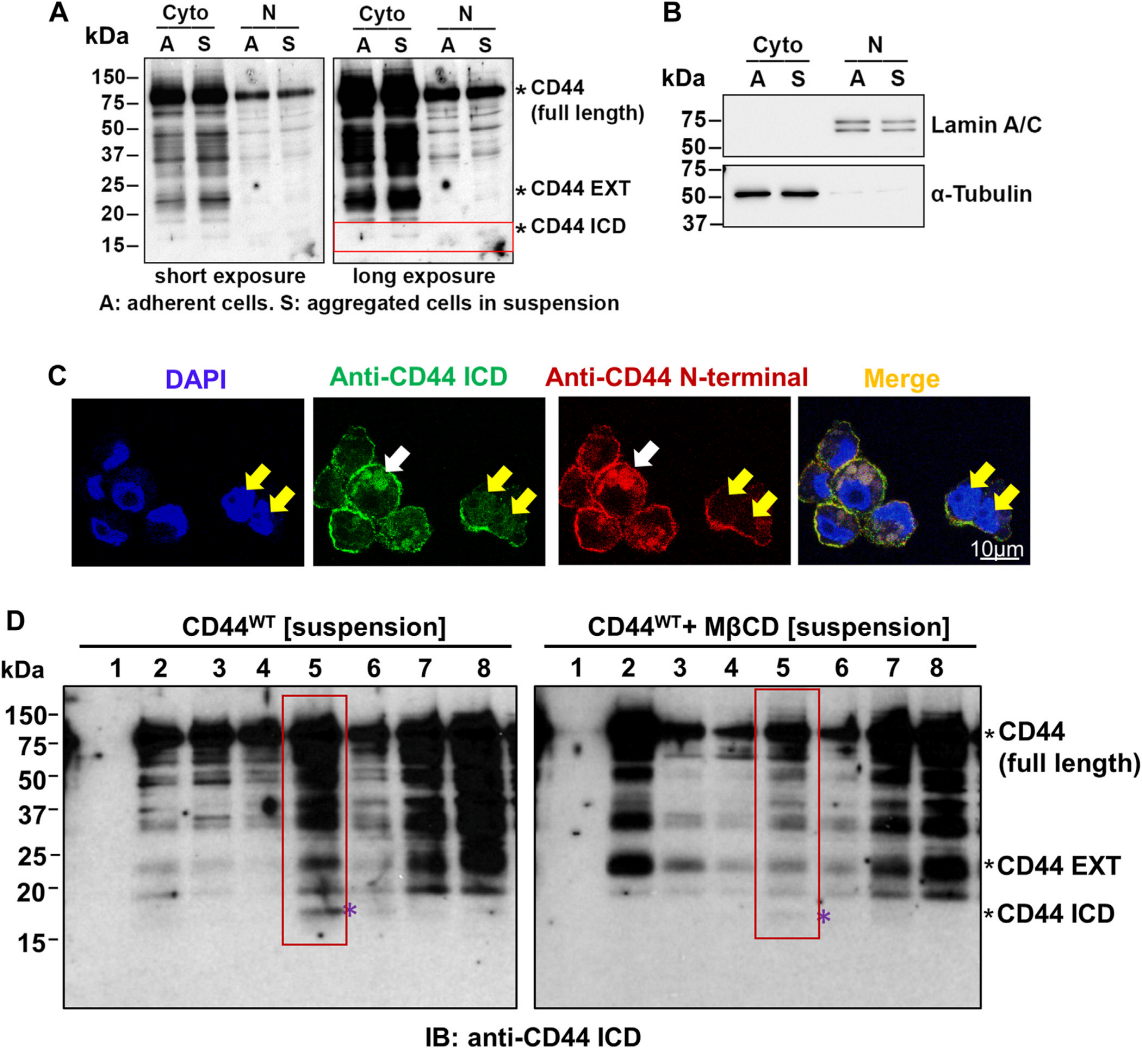
**Cell aggregation promotes CD44 cleavage to generate CD44 ICD.***A*, CD44 ICD was increased in aggregated MDA-MB-231 cells after suspension culture in poly-HEMA-coated dishes for 24 h. The cytoplasmic (Cyto) and nuclear (N) fractions were extracted for Western blotting analysis using a specific anti-CD44 antibody that can detect CD44 ICD (17 kDa). The CD44 ICD was indicated in the *red box*. CD44 EXT refers to CD44 extracellular fragment. *B*, the purity of cytoplasmic and nuclei fractions was confirmed by α-tubulin and Lamin A/C expression, respectively. *C*, representative IF staining shows CD44 ICD is localized in both cytosol (pointed with *white arrow*) and nuclei (pointed with *yellow arrows*). *D*, CD44 ICD is mainly generated in lipid rafts, but lipid raft disruption by MβCD reduces CD44 ICD generation. MDA-MB-231 cells were cultured in poly-HEMA-coated dishes in the absence of presence of MβCD (10 mM) for 2 h. The lipid rafts were isolated using Caveolae/Rafts Isolation Kit, and then used for Western blotting analysis. The fracture 5 containing CD44 ICD was shown in *red box*, and indicated with *purple star*.

Proteolytic cleavage of CD44 is a key regulatory mechanism for the CD44-dependent signaling pathways (19, 20), leading to the release of CD44 ICD, which then is translocated into the nucleus to upregulate cancer stemness-related genes such as Oct4 (24, 25, 26). Since we have found that cell aggregation upregulates Oct4 expression (11), we determined whether CD44 cleavage is induced by cell aggregation. Using a specific CD44 ICD antibody (Cosmo Bio), we found that CD44 ICD is increased in aggregated cells in both cytoplasm and nuclei (Fig. 4, *A* and *B*). The IF staining further confirmed that CD44 ICD is present in aggregated cells (Fig. 4*C*). In addition to CD44 ICD, we also observed nuclear localization of CD44 but at a significantly lower abundance than the membrane-bound CD44 (Fig. S3), consistent with previous studies (40, 41, 42). Interestingly, CD44 ICD was mainly detected in lipid rafts-enriched fraction 5 from aggregated cells. However, lipid raft disruption by MβCD dramatically reduces CD44 ICD generation in fraction 5 (Fig. 4*D*, red box). These data suggest that lipid raft integrity in aggregated cells promotes CD44 ICD generation.

### CD44 and γ-secretase coexist at lipid rafts in aggregated cells but are delocalized by lipid raft disruption

**Figure 5:**
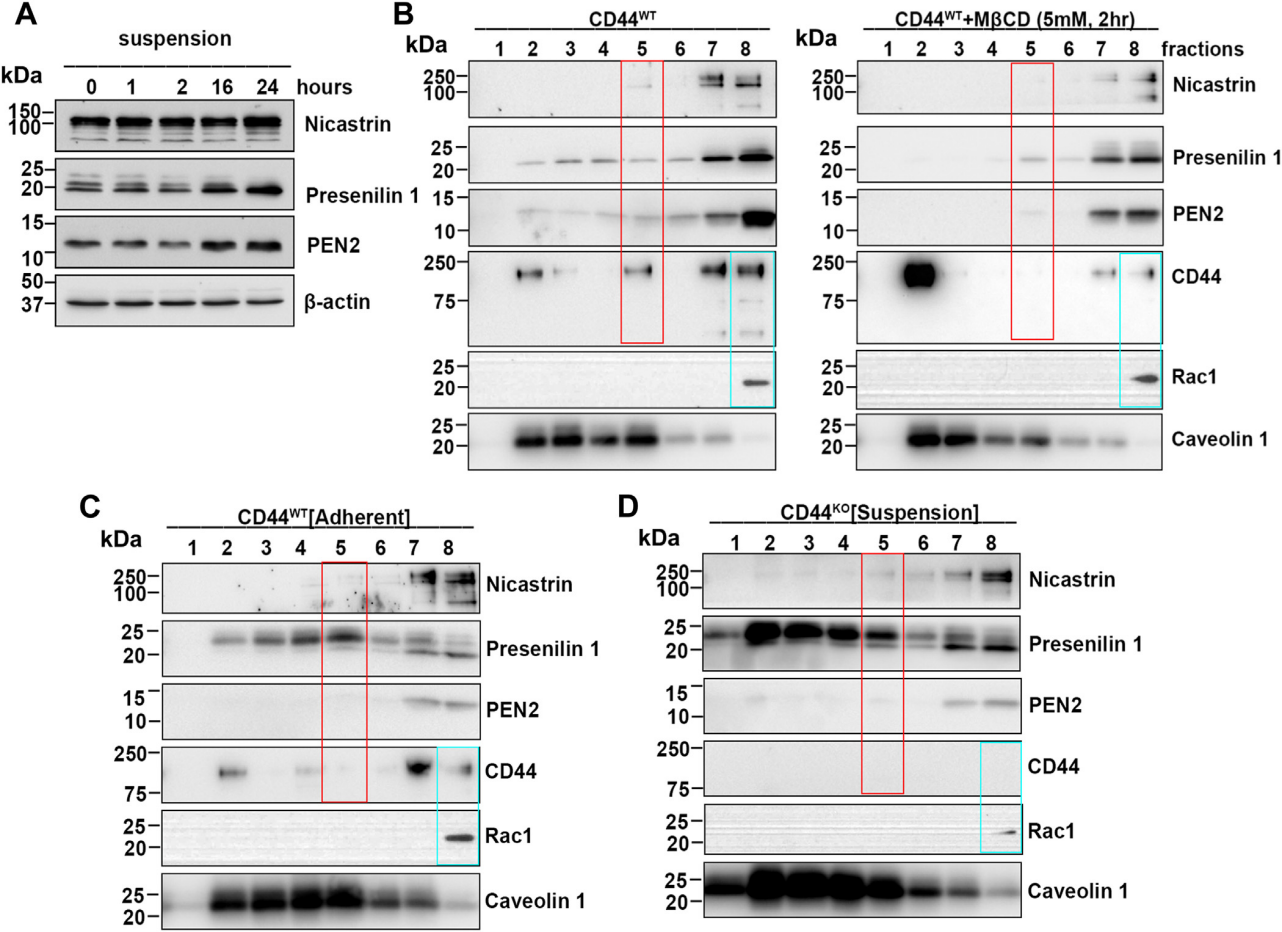
**CD44 and γ-secretase coexist at lipid rafts in aggregated cells, but are delocalized by disruption of lipid rafts.***A*, the expressions of γ-secretase complex are increased during cell aggregation over time. The MDA-MB-231 cells were cultured in poly-HEMA-coated dishes for the indicated time points, and then collected for the Western blotting analysis. *B*, CD44 and γ-secretase complex coexist at lipid rafts but are delocalized by lipid raft disruption. The MDA-MB-231 cells were cultured in poly-HEMA-coated dishes to aggregate for 24 h. To disrupt lipid rafts, aggregate cells were treated with MβCD for the last 2 h before collection. The lipid rafts were isolated using Caveolae/Rafts Isolation Kit, and then used for Western blotting analysis. *C*, the expression of CD44, γ-secretase, and Rac1 in adherent cultured CD44^wt^ MDA-MB-231 cells. The CD44^WT^ MDA-MB-231 cells were adherent cultured for 24. The lipid rafts were isolated using Caveolae/Rafts Isolation Kit and then used for Western blotting analysis. *D*, the expression of CD44, γ-secretase, and Rac1 in suspension-cultured CD44 ^KO^ MDA-MB-231 cells. The CD44^KO^ MDA-MB-231 cells were suspension cultured in poly-HEMA-coated dishes for 24 h. The lipid rafts were isolated using Caveolae/Rafts Isolation Kit, and then used for Western blotting analysis. The expression of CD44 and γ-secretase complex at fraction 5 was indicated in *red box*, and the expression of CD44 and Rac1 at fraction 8 was shown in a *blue box*. All data represent one of two independent experiments.

CD44 is cleaved by matrix metallopeptidases followed by γ-secretase to generate CD44 ICD (19, 20, 21, 22, 23, 24). We wondered whether γ-secretase could be involved in CD44 ICD generation during cell aggregation. Since γ-secretase is a multiprotein complex comprised of Presenilin, Nicastrin, Aph-1, and PEN2, all of which are essential for complete proteolytic activity, whereas the absence of even one results in the absence of γ-secretase activity (43, 44, 45), we measured the expression of these proteins using γ-Secretase Antibody Sampler Kit (Cell Signaling Technology). Indeed, Presenilin, Nicastrin, and PEN2 were increased in aggregated cells over time (Fig. 5*A*). It is known that activated γ-secretase is predominantly localized in lipid rafts (29, 46, 47). To further determine how γ-secretase is involved in CD44 ICD generation, we isolated lipid rafts from suspension-cultured MDA-MB-231 cells treated with or without MβCD, and examined the expression of γ-secretase complex by Western blot. Strikingly, CD44 and γ-secretase complex were co-expressed in lipid rafts-enriched fraction 5 in aggregated cells, but lipid raft disruption by MβCD completely delocalized their co-expression in fraction 5, accompanied with CD44 moved to fraction 1, and Presenilin, Nicastrin, and PEN2 mainly detected in faction 7 and 8 (Fig. 5*B*, red box). It is worth noting that the co-expression of CD44 and γ-secretase complex in fraction 5 was not observed in adherent-cultured MDA-MB-231 cells (Fig. 5*C*). In addition, the localization of Nicastrin and PEN2 in fracture 5 was dramatically reduced in suspension-cultured CD44^KO^ MDA-MB-231 cells (Fig. 5*D*). Furthermore, in MCF10A cells, a non-tumorigenic human mammary epithelial cell line, we observed a diminished ability for cell aggregation (Fig. S4*A*). Consequently, although CD44 was colocalized with Caveolin-1 in fractions 4 and 5, the presence of presenilin1 and PEN2 in fraction 5 of MCF10A cells was absent (Fig. S4*B*). These data suggest that the coexistence of CD44 and γ-secretase in lipid rafts is specially induced during CD44-mediated cell aggregation in anoikis-resistant metastatic cells. Considering that generation of CD44 ICD in fraction 5 was inhibited by lipid raft disruption (Fig. 4*D*), these data suggest that lipid raft integrity is necessary for the coexistence of CD44 and γ-secretase components in fraction 5 of lipid rafts, promoting CD44 cleavage and generating CD44 ICD.

### Disruption of lipid rafts inhibits the metastatic ability of aggregated cells

**Figure 6:**
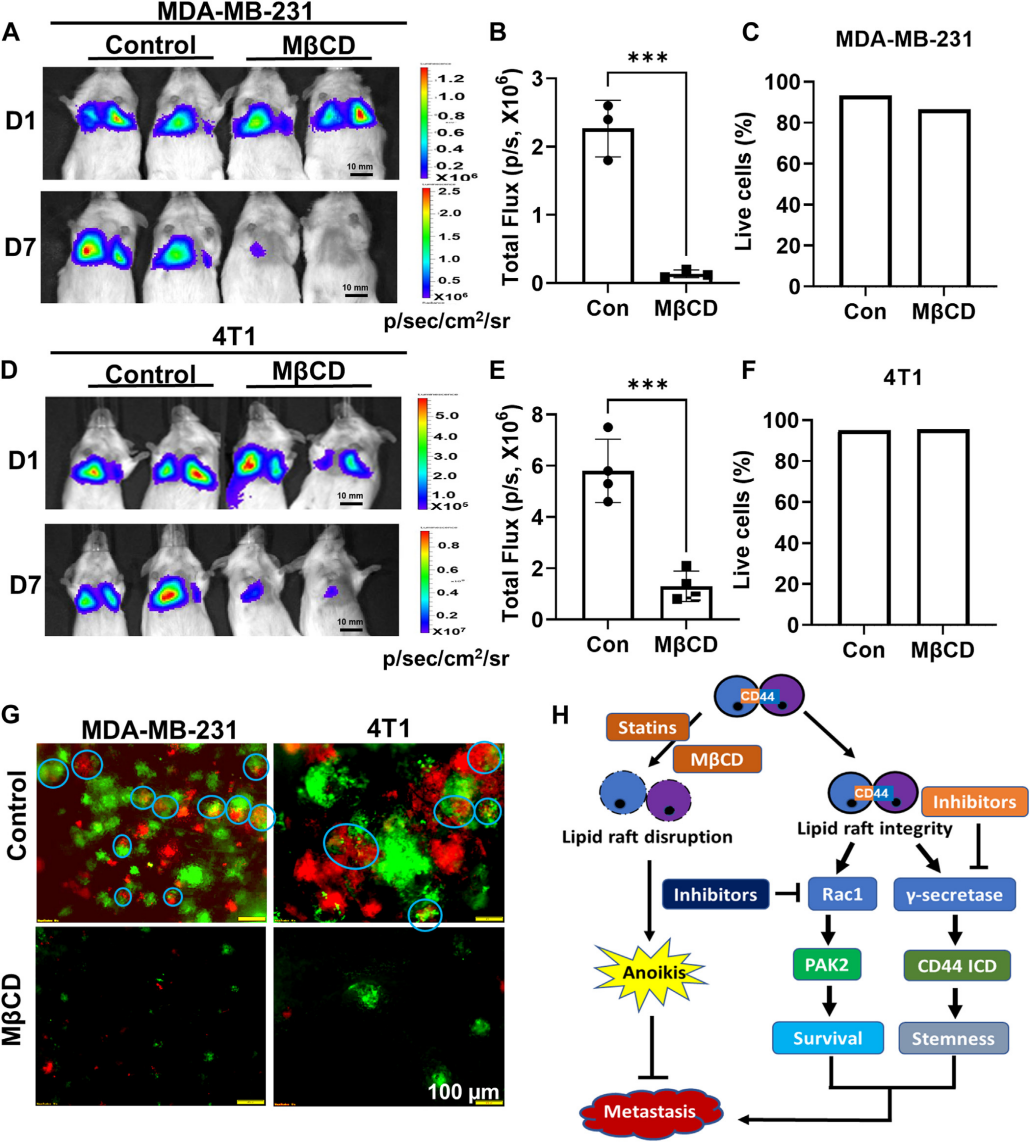
**Disruption of lipid rafts inhibits metastatic ability of aggregated cells.***A*–*C*, mixture of L2T and L2G-labelled CD44^WT^ MDA-MB-231 TNBC cells (1:1 ratio) were cultured in poly-HEMA-coated dishes with or without 5 mM MβCD for 2 h before being injected into the NSG mice *via* tail vein. The representative BLI images of lung metastases were shown (*A*), and lung metastases at Day 7 (D7) were quantitated (*B*). Graph data were presented as mean ± SD with n = 4 mice/group. *t-*test, ∗∗∗*p* < 0. 001. Cell viability before injection was measured by Vi-CELL BLU cell viability analyzer, and the average of replicates was shown (*C*). *D*–*F*, mixture of L2T and L2G-labelled CD44^WT^ 4T1 TNBC cells (1:1 ratios) were cultured in poly-HEMA-coated dishes with or without 5 mM MβCD for 2 h before injected into Balb/c mice *via* tail vein. The representative BLI images of lung metastases were shown (*C*), and lung metastases at Day 7 (D7) were quantitated (*D*). Graph data were presented as mean ± SD with n = 4 mice/group. *t-*test, ∗∗∗*p* < 0.001. Cell viability before injection was measured by Vi-CELL BLU cell viability analyzer, and the average of replicates was shown (*F*). *G*, representative immunofluorescence images of lung metastasis of mice from (*A*) and (*D*). The mixed-color polyclonal metastases were shown in *blue circle*. *H*, diagram of CD44-mediated cell aggregation maintains lipid raft integrity to prevent anoikis and enhance stemness to promote metastasis.

Cell aggregation *via* CD44 homophilic interaction after cell detachment enhances the metastatic ability of aggregated cells (11, 12). To further understand how cell aggregation promotes TNBC metastasis, we tested the effect of lipid raft disruption on the metastatic ability of aggregated cells. In order to show the mixed-color polyclonal metastases generated from aggregated cells, the L2T (red) and L2G (green)-labeled cells were mixed at 1:1 ratio, as we described previously (11). The mixed cells were cultured in poly-HEMA-coated dishes with or without MβCD for 2 h for aggregation before being injected into mice *via* tail vein. The metastatic ability of MβCD-treated cells measured by BLI was significantly reduced compared with the control group in both MDA-MB-231 and 4T1 models (Fig. 6, *A*–*F*). To rule out the possibility that reduced metastasis is a result of increased cell death, we assessed cell viability before injection, and there was no significant difference between each group (Fig. 6, *C* and *F*). In the end, the lung was removed and subjected to fluorescence imaging to directly visualize the mixed-color polyclonal metastases generated from aggregated cells (Fig. 6*G*). These data indicate that the lipid raft integrity in aggregated cells is important for the enhanced metastatic ability of aggregated cells.

## Discussion

We recently found that cell aggregation *via* CD44 homophilic interaction enhances stemness, survival, and metastatic ability of TNBC cells (11, 33). Here, we further investigated the mechanism underlying the enhanced metastatic ability of aggregated cells. Our findings suggest that CD44-mediated cell aggregation maintains lipid raft integrity after cell detachment. This, in turn, activates Rac1 to prevent anoikis of aggregated cells, and generates CD44 ICD to enhance stemness of aggregated cells (Fig. 6*H*). These data provide a new insight into the cell aggregation-mediated metastasis, and a potential therapeutic strategy to especially prevent CTC cluster-initiated TNBC metastasis by disrupting lipid raft integrity and blocking its-mediated downstream pathways such as Rac1 activation and CD44 cleavage.

Lipid rafts serve as a hub for the initiation of cellular signaling pathways by organizing the pathway components in ordered microdomains on the cell surface (7, 8, 9). Cell detachment induces internalization of some lipid raft-related components, such as caveolin and cholesterol, and changes the activities of a variety of signaling molecules (48). Consistently, our data showed that caveolin-1 was internalized into the cytoplasm in single cells after cell detachment. However, caveolin-1 was maintained on cell membrane in aggregated cells. Anoikis is activated upon cell detachment. We previously found that CD44-mediated cell aggregation prevents cells from anoikis after cell detachment (11). Here, we further demonstrated that lipid raft disruption, either by MβCD or simvastatin promotes anoikis of aggregated cells. These data strongly suggest that lipid rafts integrity in aggregated cells is important for their survival. Simvastatin belongs to a group of drugs called “statins”, which lower cholesterol levels (49). Several retrospective clinical studies have shown that statins reduce cancer mortality, recurrence, and metastasis (50, 51, 52, 53, 54, 55). Our findings reveal a new mechanism about how statins can inhibit cancer metastasis, and suggest that statins could be more efficient in eliminating CTC cluster-initiated metastasis. Future clinical trials are warranted to compare the response to statins between cancer patients with or without CD44^+^ CTC clusters. It is worth noting that lipid raft disruption also induces apoptosis in adherent cells (56). Consistently, MβCD treatment induced apoptosis (Annexin V+ cells) in both adherent MDA-MB-231 and 4T1 cells (Fig. S5) but to a less extent than in non-adherent cells. However, in contrast to its effect on detached cells (Fig. 3), MβCD did not reduce but rather slightly increase Rac1 activity in adherent cells (Fig. S6). Collectively, our finding raises a possibility that disruption of lipid rafts induces cell death in adherent cells and non-adherent cells *via* different pathways and mechanisms, which warrants further investigation.

It has been reported that cell aggregation inhibits anoikis after ECM-detachment in Her2 (ErbB2)-positive breast cancers (34). In the study, it is reported that cell aggregation induces EGFR stabilization, and consequently activates the ERK survival pathway in an E-cadherin-dependent manner in Her2-positive breast cancer cells (34). However, we did not observe ERK activation in CD44-mediated cell aggregation in the TNBC cells. Since we have known that the MDA-MB-231 TNBC cell line does not express E-cadherin (11, 57), it could explain why ERK is not activated in these cells. Interestingly, we also found that CD44 stabilizes EGFR in TNBC cells (11), and CD44-mediated cell aggregation can directly activate EGFR (33). These data suggest that anoikis resistance in aggregated CD44^+^ TNBC cells is independent of the EGFR-ERK survival pathway. Instead, we found that disruption of lipid rafts activates p38, whose activation promotes anoikis (35, 36). In addition, studies have shown that p38 induces EGFR endocytosis for its degradation (58, 59). Taken together, these data raise a possibility that lipid raft disruption activates p38, which then promotes EGFR endocytosis to inhibit its activation and downstream survival pathways. It is well-accepted that p38 promotes tumor, and the p38 inhibitors are undergoing clinical trials to treat cancer patients. However, studies have also shown that p38 activation in breast cancer cells inhibits metastasis (60). Nevertheless, it should be cautious that these inhibitors may increase the chance of metastasis by inhibiting CTC anoikis.

Drug repurposing and a combination of already approved drugs provide the quickest and lowest cost for clinical development and implementation of anti-cancer drugs. Ketorolac is approved by the FDA for the treatment of moderate to severe pain. Recent studies have identified that racemic ketorolac (R-Ketorolac) is a robust Rac1 inhibitor that inhibits ovarian cancer metastasis (38, 39). A retrospective study also found that R-Ketorolac treatment improved the survival of patients undergoing conservative breast cancer surgery (61). γ-secretase inhibitors (GSIs) have been developed to inhibit γ-secretase cleavage in humans since γ-secretase was identified as a therapeutic target in Alzheimer's disease (AD). However, side effects were observed during clinical trials, which led to the discontinuation of the development of GSIs as anti-AD drugs. One reason causing these side effects is because γ-secretase also cleaves Notch (44). Since the dysregulated Notch signaling pathway has been directly linked to multiple cancers including TNBC (62, 63, 64, 65), GSIs are being actively repurposed as anti-cancer drugs. Although GSI monotherapy shows limited clinical benefit in cancer patients, encouraging data show that the combination of GSI with chemotherapies has a synergistic effect (66, 67, 68). In this study, we found that disruption of lipid rafts inhibited both Rac1 activation and CD44 ICD generation in aggregated cells. Importantly, lipid raft disruption significantly reduced the metastatic ability of aggregated cells. MβCD has been tested as an anti-cancer drug, but only in the preclinical development stage (30, 69). Since GSI and R-Ketorolac are available FDA-approved drugs, it warrants further study to determine whether a combination of GSI and R-Ketorolac can have a better anti-metastatic effect than their monotherapy, particularly in patients with CD44^+^ CTC clusters.

## Conclusion

CTCs have become one of the major focuses of translational cancer research due to their easy accessibility for “liquid biopsy” with dynamic and live information on disease status. The presence of CD44^+^ CTC clusters correlates with a poor prognosis in breast cancer patients. A deeper understanding of the mechanism and signaling pathways mediating cell aggregation will lead to the development of new strategies to target CTC clusters and block cancer metastasis.

## Experimental procedures

### Breast cancer mouse models and animal studies

Eight to 10-week-old female NSG (NOD SCID gamma), and Balb/c mice were purchased from Jackson Lab. All mice were housed in the specific pathogen-free facilities, with normal chow diets and 12:12 h light–dark cycle at 22 °C in DLAR (Division of Laboratory Animal Resources) at the University of Kentucky. All of the animal protocols were approved by the Institutional Animal Care and Use Committee.

The human MDA-MB-231 breast cancer cell line and mouse 4T1 breast cancer cell line were labeled with L2G and L2T as described previously (11). A mixture of L2T and L2G-labelled cells (1:1 ratio) were cultured in poly-hydroxyethyl methacrylate (poly-HEMA, Sigma-Aldrich)-coated dishes for 24 h, and then treated with or without 5 mM Methyl-β-cyclodextrin (MβCD, Sigma-Aldrich, C4555) for another 2 h before being injected into the mice *via* tail vein. Lung metastasis was analyzed by using Bioluminescence imaging (BLI) as previously described (70). At the endpoint, the lungs were removed, and lung metastases were imaged by fluorescence microscopy.

### Cell culture and treatment

MDA-MB-231 and 4T1 cells were obtained from ATCC. Cells (<20 passages) were maintained in DMEM high glucose with 10% FBS + 1% penicillin-streptomycin at 37 °C in an atmosphere of 5% CO_2_. CD44 KO MDA-MB-231 cells were generated as described previously (11). For suspension cell culture, cells were trypsinized into single cells and then seeded to poly-HEMA-coated plates and treated with or without MβCD, simvastatin (Sigma-Aldrich, S6196), and R-Ketorolac (MedChemExpress, HY-B0580B).

### Caveolae/rafts isolation

Lipid raft fractions were isolated by a commercial caveolae/rafts isolation Kit (Sigma-Aldrich, CS0750) according to the instructions. Briefly, cells were washed twice with ice-cold PBS and lysed using lysis buffer containing 1% Triton X-100 and 1% protease inhibitor cocktail for 30 min on ice. The density gradients were prepared at 35%, 30%, 25%, 20%, and 0% concentrations using the recommended amounts of the cell lysate, lysis buffer, and OptiPrep medium and then centrifuged at 200,000*g* for 4 h at 4 °C using a TH-641 rotor (WX+ ultracentrifuge, Thermo Scientific). Each fraction was carefully collected from top to bottom of the ultracentrifuge tube and transferred to a microcentrifuge tube. The fractions were analyzed by Western blot.

### Extraction of nuclear and cytoplasmic fractions

Cells were suspension cultured in poly-HEMA-coated dishes for 24 h, and then the nuclear and cytoplasmic fractions were extracted using a commercial nuclear extraction kit (Active Motif, 40010) according to the manufacturer’s instructions, followed by Western blotting analysis. The purity of cytoplasmic and nuclei fractions was confirmed by α-tubulin and Lamin A/C expression, respectively.

### Western blotting

Cells were lysed by RIPA buffer with Halt Protease Inhibitor Cocktail (Thermo Fisher, 78430) for 30 min on ice, then centrifuged for 12 min at 4 °C. Protein concentration was measured through BCA Protein Assay Kit (Thermo Fisher, 23227), and equal amounts of protein of each sample (10–40 μg) were separated by SDS-PAGE and then transferred to nitrocellulose membranes. After blocking with 5% non-fat dry milk in 0.1% tween/TBS (Tris-buffered saline) for 1 h, the membrane was washed and incubated with primary antibody overnight at 4 °C. The appropriate HRP-conjugated second antibody was added after washing with 0.1% tween/TBS. The transferred protein was detected using the clarity western ECL reagent (Bio-Rad, 1705061), a Bio-Rad ChemiDoc imaging system, or X-ray film. Primary antibodies used were Rac1 (1:500, Cytoskeleton, ARC03), CD44 (1:2000, Thermo Fisher, MA5-15462), CD44 ICD (1:1000, COSMO BIO USA, KAL-KO601), p-EGFR (Tyr845) (1:1000, Cell Signaling, 2231), EGFR (1:1000, Santa Cruz, sc-03), p-p38 (Thr180/Tyr182) (1:1000, Cell Signaling, 4511), p38 (1:1000, Cell Signaling, 9212), p-ERK1/2 (Thr202/Tyr204) (1:1000, Cell Signaling, 4370), ERK1/2 (1:1000, Santa Cruz, sc-514302), Caveolin 1 (1:5000, Sigma-Aldrich, C3237), Lamin A/C (1:1000, Cell Signaling, 4777), α-Tubulin (1:4000, Sigma-Aldrich, T5168), Nicastrin (1:1000, Cell Signaling, 5665), Presenilin1 (1:1000, Cell Signaling, 5643), PEN2 (1:1000, Cell Signaling, 8598), β-Actin (1:1000, Thermo Fisher, MA5-11869). Secondary antibodies used were goat polyclonal anti-mouse (IgG) HRP (1:10,000) (Thermo Fisher), and goat polyclonal anti-rabbit (IgG) HRP (1:10,000) (Thermo Fisher).

### Rac1 pulldown activation assay

Rac1 activity was carried out using a Rac1 Pull-Down Activation Assay Biochem Kit (Cytoskeleton, BK035) according to the instruction. Briefly, cells were lysed, and equivalent amounts of protein lysates were incubated with PAK-PBD beads at 4 °C for 1 h. Beads were washed with lysis buffer, and precipitated Rac1-GTP was analyzed by SDS-PAGE and Western blotting. Rac1-GTP levels were normalized to total Rac1 by densitometric analysis with ImageJ software.

### Immunofluorescence

Cells were cultured in poly-HEMA-coated dishes to aggregate for 24 h. Afterward, cells were collected and spun onto Fisherbrand Superfrost Plus microscope slides (Thermo Fisher, 22-037-246), and fixed with 4% paraformaldehyde for 10 min. Cells were permeabilized using 0.25% Triton X-100 in PBS, followed by blocking with 2% BSA in PBS for 1 h. Caveolin 1, CD44 (N-terminal), and CD44-ICD primary antibodies were then incubated with cells overnight at 4 °C. On the second day, cells were then washed and incubated with Alexa Fluor 488 and Alexa Fluor 568-conjuated secondary antibodies (Thermo Fisher) for 1 h, and finally, nuclei were counterstained with DAPI. The images were taken on a confocal microscope (Nikon Confocal).

### Anoikis analysis

Poly-HEMA was reconstituted in 95% ethanol to a concentration of 20 mg/ml. To prepare poly-HEMA-coated plates, poly-HEMA solution was added to cell culture plates/dishes and allowed to dry overnight in a tissue culture hood. Cells were cultured in poly-HEMA-coated plates/dishes as indicated time points, and the anoikis was analyzed by annexin V/PI staining (Pacific Blue Annexin V Apoptosis Detection Kit with PI; BioLegend) according to the manufacturer's instructions.

### Statistical analysis

Student's *t-*test was performed for the statistical analyses between two samples as appropriate using GraphPad Prism software. One-way ANOVA (followed by Tukey post-hoc test) was performed to analyze differences among multiple groups. Data are presented as mean ± SD from at least three biological replicates, and *p* < 0.05 was considered significant.

## Data availability

Raw data of this study are available from the corresponding author upon reasonable request.

## Supporting information

This article contains supporting information.

## Conflict of interest

No conflicts of interest, financial or otherwise, are declared by the authors.
