# Supplementary figures and images for: Cell aggregation activates small GTPase Rac1 and induces CD44 cleavage by maintaining lipid raft integrity
Source: J Biol Chem. 2023 Oct 20;299(12):105377. doi: 10.1016/j.jbc.2023.105377 (PMC10692920; doi:10.1016/j.jbc.2023.105377)

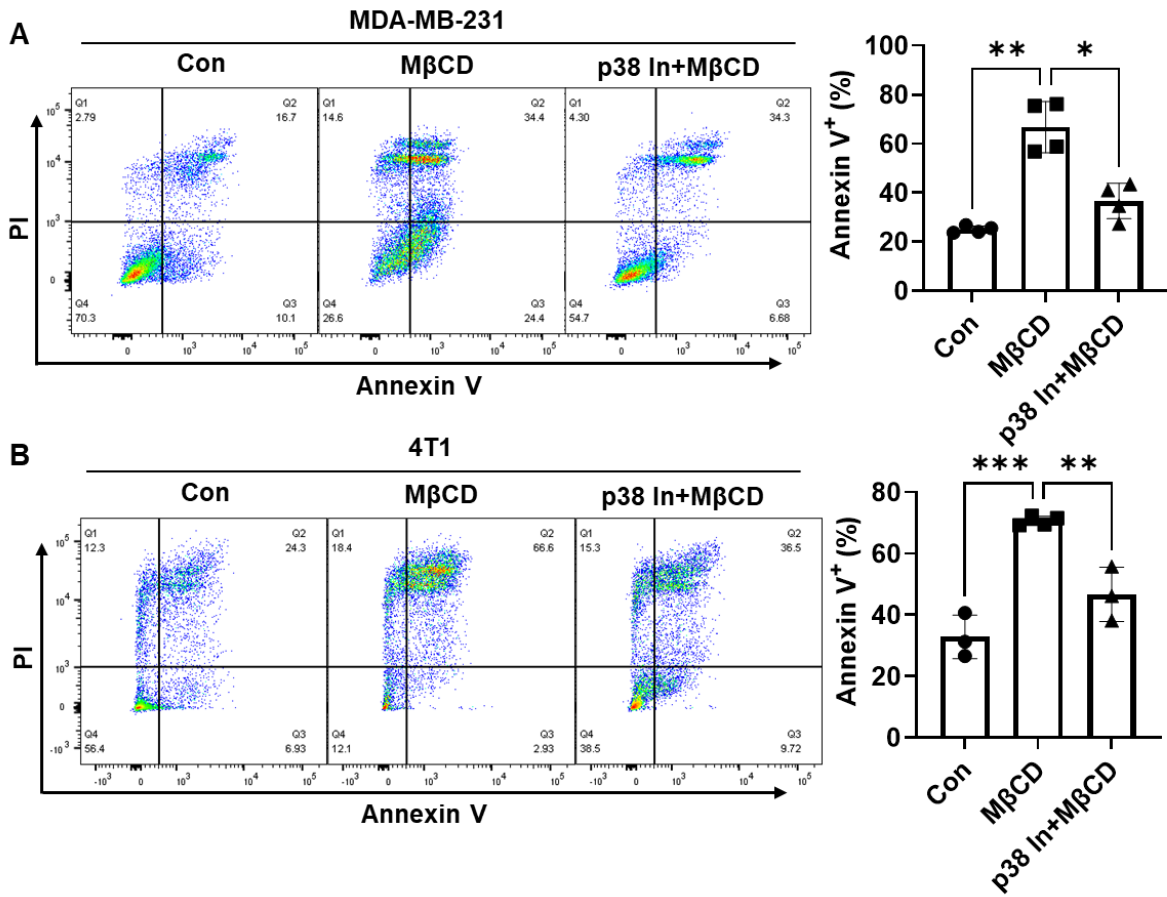

Supplement: Supplemental Figure S1 [file mmc1.pdf]

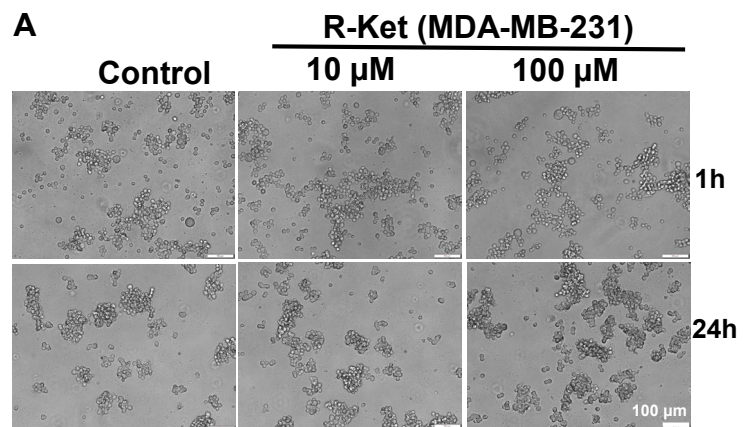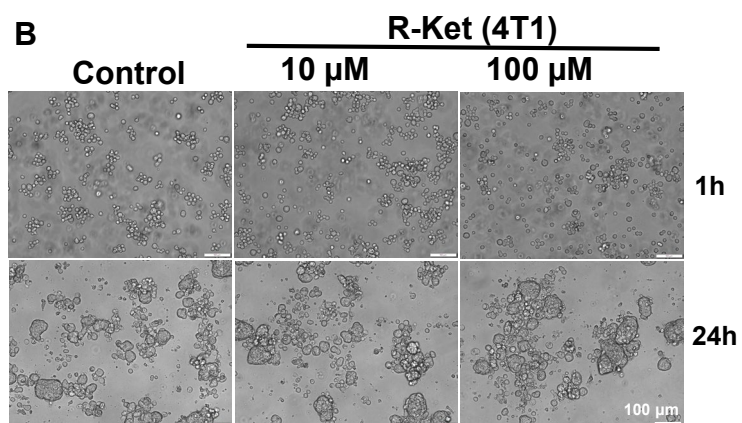

Supplement: Supplemental Figure S2 [file mmc2.pdf]

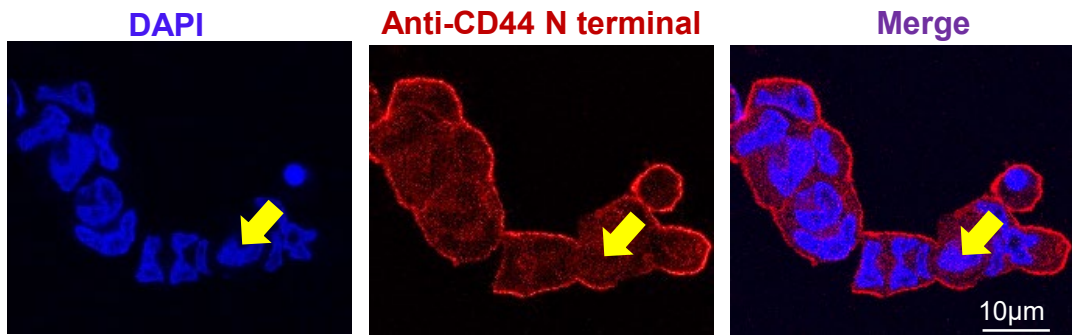

Supplement: Supplemental Figure S3 [file mmc3.pdf]

**A**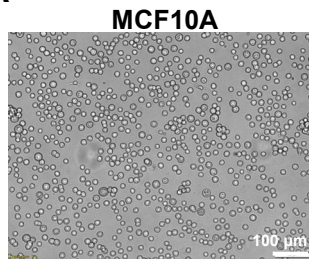**B**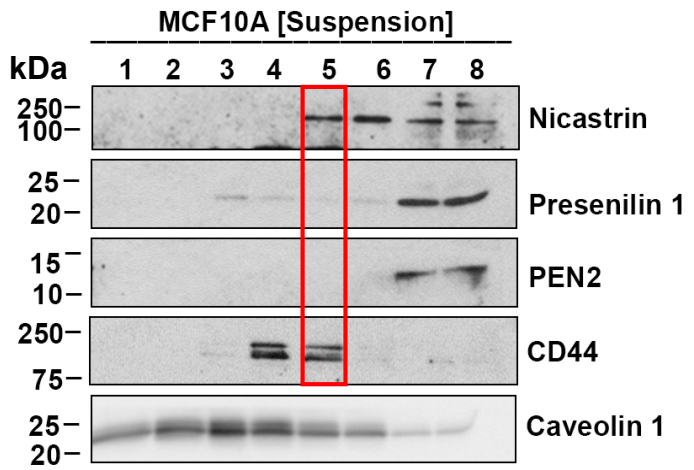

Supplement: Supplemental Figure S4 [file mmc4.pdf]

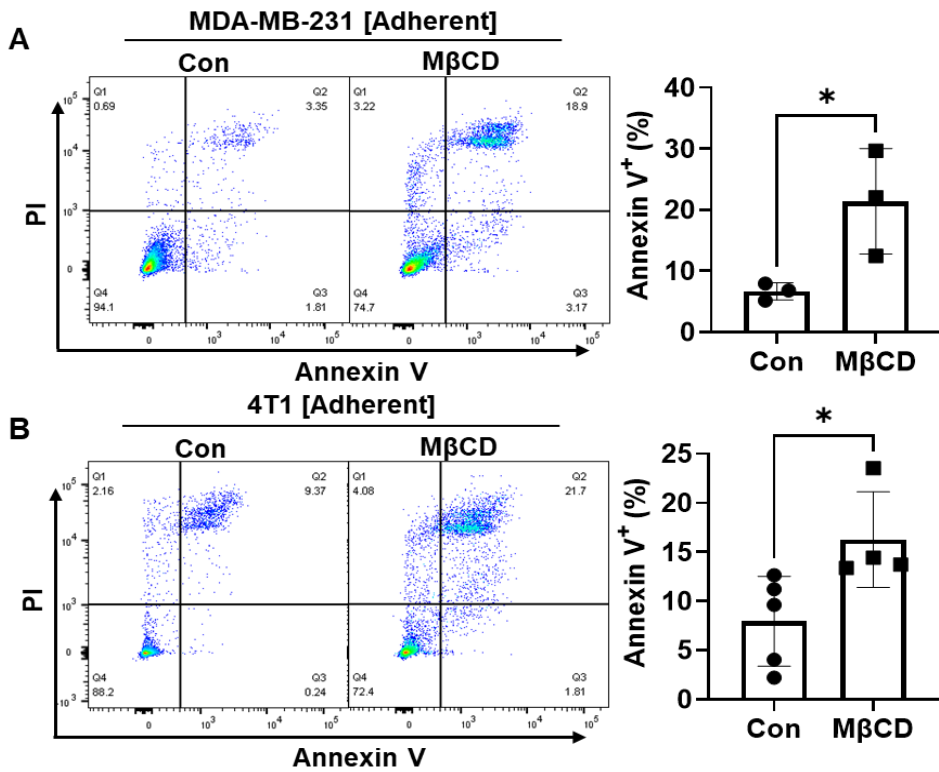

Supplement: Supplemental Figure S5 [file mmc5.pdf]

**A**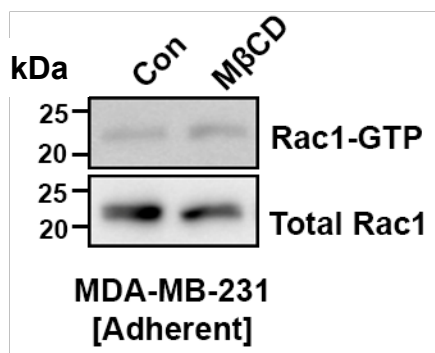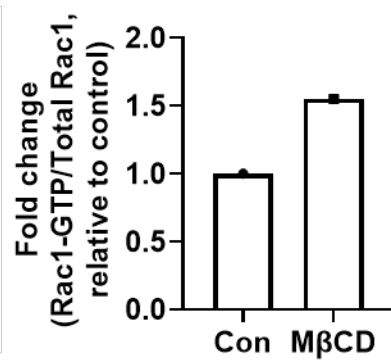**B**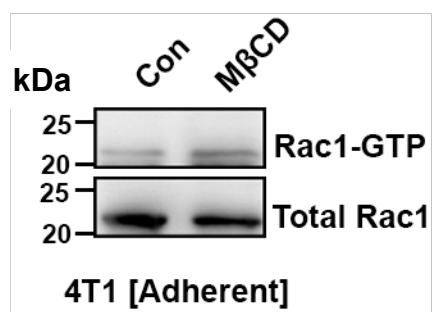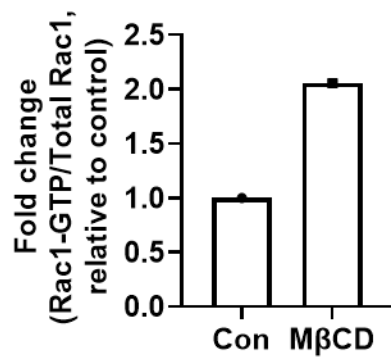

Supplement: Supplemental Figure S6 [file mmc6.pdf]
