# Supplementary material for: Cell aggregation activates small GTPase Rac1 and induces CD44 cleavage by maintaining lipid raft integrity
Source: J Biol Chem. 2023 Oct 20;299(12):105377. doi: 10.1016/j.jbc.2023.105377 (PMC10692920; doi:10.1016/j.jbc.2023.105377)
Supplement: Supplemental Figure legends [file mmc7.docx]

**Figure S1. Inhibition of p38 partially prevents anoikis.**

The MDA-MB-231 cells (**A**) and 4T1 cells (**B**) were pre-incubated with or without p38 inhibitor (SB203580 1µM) for 1 hour, and then cultured in poly-Hema-coated dishes with or without MβCD (5mM) for 24 hours. The cells were collected for anoikis analysis by Annexin V/PI staining (left). The percentage of Annexin V+ cells were quantitated (right). Graph data were presented as mean ± SEM (n=3-4). One-way ANOVA, *p<0.05, **p<0.01, ***p<0.001.

**Figure S2. R-ket does not inhibit cell aggregation in MDA-MB-231 or 4T1 cells.**

**A**. Representative images of MDA-MB-231 cells cultured in poly-Hema-coated dishes in the presence or absence of R-Ketorolac for the indicated time points. **B**. Representative images of 4T1 cells cultured in poly-Hema-coated dishes in the presence or absence of R-Ketorolac for the indicated time points.

**Figure S3. Nuclear localization of CD44.**

Representative IF staining shows CD44 (determined by an anti-CD44 antibody reacts with the extracellular N-terminal domain of CD44) is localized in nuclei (pointed with yellow arrows).

**Figure S4. Localization of CD44 and γ-secretase components in MCF10A cells.**

**A**. Representative image showing MACF10A cells are not aggregated after culture in poly-Hema-coated dishes for one hour. **B**. The MCF10A cells were cultured in poly-Hema-coated dishes for 24 hours, the lipid rafts were isolated using Caveolae/Rafts Isolation Kit, and then used for western blotting analysis. The localization of CD44 and γ-secretase components in fracture 5 was shown in red box.

**Figure S5. The effect of lipid rafts disruption on adherent cells.**

The adherent cultured MDA-MB-231 cells (**A**) and 4T1 cells (**B**) were treated with or without MβCD (5mM) for 24 hours, and then the cells were collected for Annexin V/PI staining (left). The percentage of Annexin V^+^ cells were quantitated (right). Graph data were presented as mean ± SEM (n=3-5). t-test, *p<0.05.

**Figure S6. The effect of lipid rafts disruption on Rac1 activation in adherent cells.**

The levels of active Rac1-GTP in adherent MDA-MB-231 cells (**A**) and adherent 4T1 cells (**B**) are normalized with the levels of total Rac1 protein in cells, and the fold changes compared with control are calculated (right). The data represent one of two independent experiments.
